# The Importance of Numeric Databases to Materials Science

**DOI:** 10.6028/jres.094.002

**Published:** 1989

**Authors:** Richard A. Matula

**Affiliations:** AT&T Bell Laboratories, 600 Mountain Avenue, Murray Hill, NJ 07974

**Keywords:** CRYSTDAT, industrial use of numeric databases, lattice matching, materials science, numeric databases, online searching, prediction of ferroelectricity, superconductivity

## Abstract

Scientific numeric databases are important research tools for materials scientists. In distinction to bibliographic databases, these numeric databases are useful primarily to provide direct, immediate access to data, often evaluated data. Examples showing the application of crystallographic databases are given, including determining candidate materials for certain applications. Thermo-chemical data useful for optimizing optical fiber processing are discussed showing the importance of high-quality data. In addition, these databases are an important tool that can be utilized in the graduate education of the next generation of materials scientists.

## Introduction

In this introduction, I would like to briefly point out some aspects of the importance of numerical databases to materials sciences, specifically the importance of good data and the quick dissemination of numeric data to a wide audience. All the examples that are mentioned come from the work at AT&T Bell Laboratories.

If one were to claim that numeric databases were the most important development in materials science, that would clearly be an overstatement. On the other hand, if one were to claim that they are of no value to materials science, that would be understating their importance. The importance lies in between and can be expected to increase as knowledge of them becomes more widely known. Many of these databases, specifically in the crystallographic area, have developed from print products that existed for decades and putting them into numeric databases is a shift in content and in means of access. But it’s more than that, much more than that. As with many new technologies, there are uses that are unanticipated when a new technology first appears. The usual advantages of having data in numeric form include the following: they’re easy to update, there’s a potentially faster delivery of information to the user, and there’s a potentially large audience of scientists and engineers that can be reached by having the data in the electronic form, because these people can access this data by their personal computer or microcomputer in the laboratory, the office, or even at home.

I want to point to several examples. First, I’ll discuss one of the main processes for making optical fibers, which I’ll spend the most time on, and which will illustrate the need for good data. Other examples I will mention are the prediction of ferroelectricity, lattice matching, and high-*T*_c_ super-conductivity. I’ll close with a lesson from history.

## Modified Chemical Vapor Deposition (MCVD) for Optical Fibers

This first example^1^ will show the crucial importance of good data to industry. This concerns modeling the process involved in the manufacture of optical fibers.

**Figure 1:**
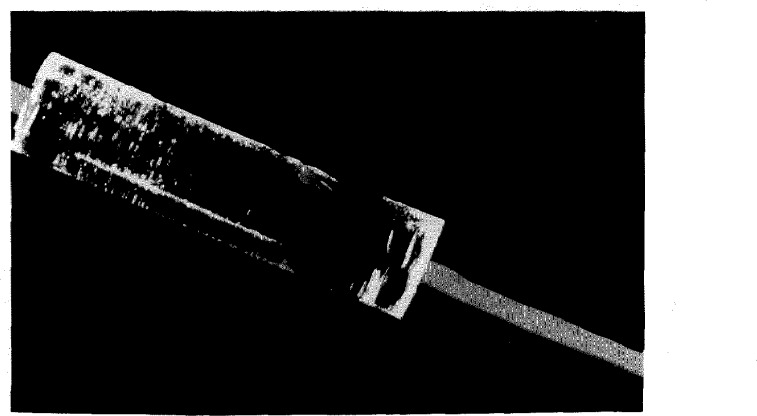
Scattering not observed in highly purified piece of glass rod.

Figure 1 shows a part of a highly purified piece of glass rod that would be used to make an optical fiber. One can observe hardly any scattering coming from the laser beam (which goes from the upper left to the lower right) going through the glass rod. This is due to both the high purity and the absence of scattering centers. There is a high index of refraction core in the center of the rod. This core is formed by doping silica glass with germanium and phosphorus. When an optical fiber is drawn from the rod, this core serves to guide the laser beam as it travels through the fiber. The rod or preform shown in figure 1 was made by a process known as the MCVD process developed by J. B. MacChesney and others at AT&T Bell Laboratories in the mid-1970s [3]. Two other processes have been developed to produce similar fibers; they are the OVD (outside vapor deposition) process, otherwise known as the Corning process, and the VAD (vapor phase axial deposition) process, which is mainly used in Japan.

**Figure 2:**
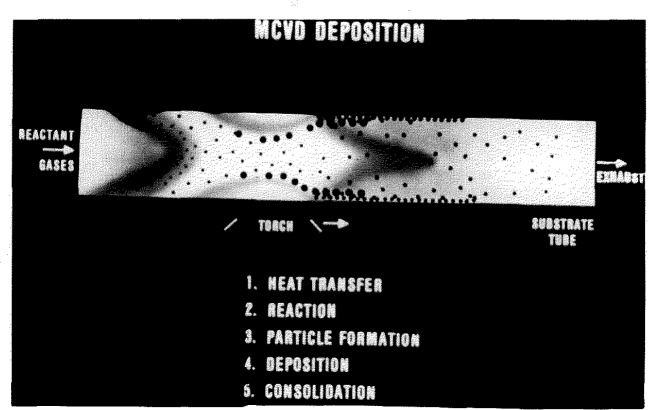
Essence of MCVD process.

Figure 2 is a schematic representation of MCVD. This shows a silica tube being rotated in a lathe. Reactant gases enter on the left, pass through, and are exhausted. An oxyhydrogen torch heats the glass tube together with the reactant gases inside, and the torch travels slowly the length of the tube in the direction indicated. In the heated zone (about 1650 K), silicon tetrachloride and other halides react with oxygen to form oxide particles, and slightly downstream these particles deposit on the inside wall of the tube [4] and are vitrified as the torch passes over them. The tube will later be collapsed and then drawn into a hair-thin optical fiber many kilometers long.

**Figure 3:**
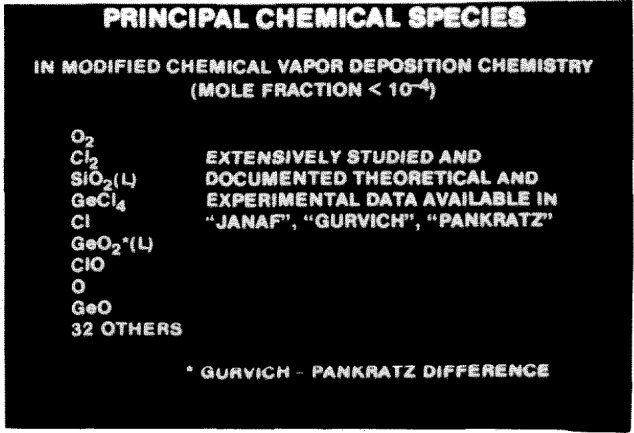
Principal chemical species in MCVD chemistry.

Figure 3 shows the principal species involved in the reactions in the heated zone, namely SiO_2_, GeO_2_, etc., together with 32 gaseous and liquid species. Thermochemical data for these species needed for modeling the process came from several sources [5,6,7,8]. The existence and concentration of these species were imperfectly known in the minds of researchers for some time. Empirical work, together with modeling, led to a better understanding of the process including better values of equilibrium constants [9]. What is important is the formation of GeO_2_ from GeCl_4_. Both species are present at equilibrium and small changes in deposition conditions can significantly change the concentration of each.

**Figure 4:**
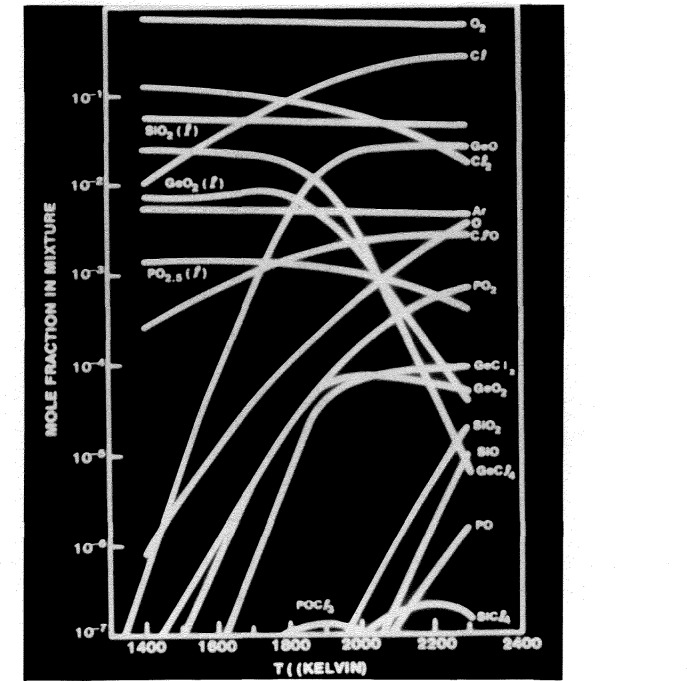
Mole fraction of chemical species in MCVD process.

**Figure 5:**
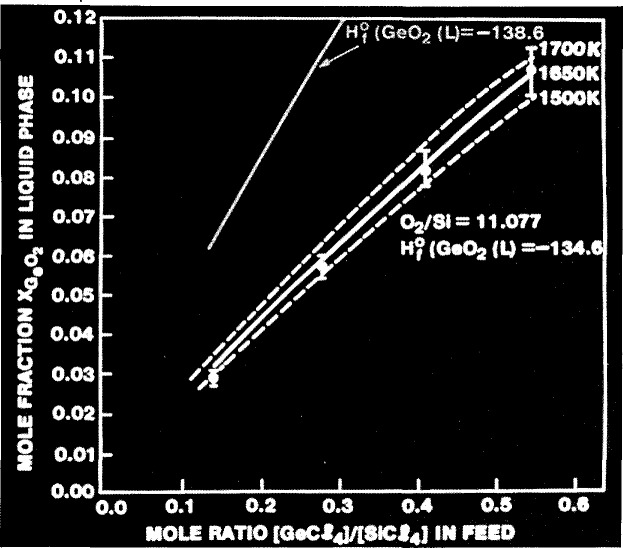
Difference of 4 kilocalories in heat of formation of germanium dioxide important for modeling MCVD process.

Figure 4. shows the mole fraction of the various reactants and products as a function of temperature. The bump in the GeO_2_ curve was predicted by the modeling of the process and was later verified experimentally. Figure 5 shows the mole fraction of GeO_2_ in the liquid phase as a function of the Ge/Si ratio in the feed gas. The two dotted lines and the solid line in between come from the model calculations for various temperatures. The dots and error bars come from experimental data. The heat of formation that was chosen was such as to get the best agreement between the modeling and the experimental data. However, another reputable researcher has heat of formation data, leading to the upper curve, that differs by about 4 kilocalories compared to the heat of formation data that fits the modeling and experimental data very well. This shows the need for heat of formation data to better than 1 kilocalorie instead of 4 kilocalories. A more extensive discussion of this difference appears in the literature [10].

This example illustrates that better values of data, when placed in the proper theoretical and experimental context, can lead to a more thorough understanding and an optimization of an industrial process. This optimization has further implications when it is realized that the AT&T plant in Atlanta is capable of producing a large mileage of fiber per year, and hence is of significant commercial importance.

## Predictions of Ferroelectricity

Numerical databases can be used in novel ways. The previous example would exemplify the usual retrieval of numerical data for a certain material. Ten years ago, when I was at CINDAS (Center for Information and Numerical Data Analysis and Synthesis, Purdue University), most of the requests for retrieval of data were just of this kind. For example, one would ask for resistivity of palladium over a certain temperature range. At that time we thought of the potentiality of reversing that type of search. In the reverse type of search one would ask for a list of materials fitting a certain set of conditions. For example, in the design of a spacecraft, what materials would have both a flat thermal linear expansion over a certain temperature range and a thermal emissivity between two given values? That illustrates the use of numeric databases for a new type of question which we are now able to answer, and S. C. Abrahams of AT&T Bell Labs has discussed how he utilized the Inorganic Crystal Structure Database (ICSD) mounted in Karlsruhe, West Germany, to predict ferroelectricity in materials from point group 6*mm* using a structure-based approach [11]. Using the database, together with the criterion of polar space groups, the number of structures that needed to be examined was considerably reduced; additional structural criteria were then used to make the final predictions. He points out that experimental verification of each prediction is needed. The approach of using a crystallographic database for this type of query is important and advances such forward-looking research.

## Lattice Matching

Another example of this reverse approach is that of lattice matching. If one has, for example, gallium arsenide and wants to make sure that a material for lattice matching has not been overlooked, these crystallographic databases can be searched to determine what materials have a certain lattice parameter very close to that of GaAs.

## High-*T*_c_ Superconductivity

Theo Siegrist discusses applications of the crystallographic database, CRYSTDAT, to high-*T*_c_ superconductivity research [12] with the result of a substantial time saving in determining a crystal structure. He and his colleagues were able to determine the crystal structure quickly, in about a day, compared to other groups having to spend a week or so. Clearly, these researchers were more productive because their time was used more efficiently.

It should be pointed out that information on crystallographic databases appears in *Crystallographic Databases* that has been recently published [13]. It contains a discussion of many other crystallographic databases in addition to the two referred to in this talk.

## Lesson from History

There’s a lesson from history as we look back at different types of databases, mainly bibliographic, that may be applicable to the work in numeric databases. The publisher of *Chemical Abstracts* has for a long time had an educational policy that encourages the use of their online database by students. The students get training while in graduate school, and when they come to industry, they expect to have this tool available. In the legal area, a similar practice was done for lawyers by Mead Data and their Lexis system of databases. In order to have the next generation of materials scientists use these databases to the maximum degree possible, how would the following question be answered: Is everything possible being done to make sure that the graduate schools are using these numerical databases in the training of materials scientists?

For the two examples mentioned using the crystallographic databases ICSD and CRYSTDAT, a personal password was obtained for each of the two researchers so that they could access the database at their convenience. What other actions, including advertising and publicity, can information professionals do to increase knowledge of these databases, and other numeric databases, in order to encourage increased usage and hence aid the technical staff to become more effective?

For present users, what other numerical databases are useful? What new combinations of data should be put together in existing databases? With the technology of split screens and microcomputers, is it possible to include additional computational power in the databases? I know John Rodgers (613-993-3294) of the National Research Council Canada, and Alan Mighell of NIST (301-975-6255) who are the conference organizers, are very interested in this question, and would like to know your thoughts. In general, other database producers would very much like to have comments from users, to determine how their databases should develop.

Lastly, what policies are in place, or if not, should be in place, to ensure that the critical work in database production and data evaluation is passed on to the next generation? What needs to be done so the torch, so to speak, does not go out because of retirement, death, or other untimely changes? It is important to ensure that the work that’s being done in and on these databases does not go for naught and can continue.

## Footnotes

1 For this example, I want to thank K. B. McAfee, Jr., who recently retired from AT&T Beil Laboratories. He presented this example in a talk, which is unpublished, at the Tenth CODATA Conference in Ottawa, July 1986. Credit for this work goes to K. B. McAfee, Jr., R. A. Laudise, R, S. Hozack, D. M. Gay, and K. L. Walker all of AT&T Bell Laboratories [1,2].
